# Popular Education

**Published:** 1911-08

**Authors:** 


### Popular Education

FIVE hundred and three students were graduated from the public high schools of Buffalo, last June. Twenty-five years ago, when the population was about half its present number, fifty was a fair sized class. It is probably a conservative statement that five times as many girls and boys—relatively to population—receive a high school training now, as thirty years ago.

Of any average community, about 1.8 per cent. consists of girls and boys of the age at the end of the high school course.

Counting the population of Buffalo as 425,000, there are 7,650 of this age. One in fifteen of them actually completes the academic course in the public schools. Probably, including parochial and private schools, at least one in ten is educated to this degree. About half of the rising generation has, for some years, had the benefit of what was, a generation ago, the first year of a high school course. The present full high school course covers approximately half of the college course of our grandfathers.

Buffalo is not exceptional in school attendance. Speaking broadly, the same degree of education is received in most cities, even in most villages and rural districts in most of our states, excepting some few, of relatively small population and having to deal with peculiar problems of race or sparse distribution of inhabitants.

Is this a proper topic for consideration by a medical journal? Yes, it indicates on the one hand that the raw material for recruiting the ranks of our profession is better prepared than ever before; on the other hand that the laity to whom they will minister, will insist more strongly on advanced professional standards and will co-operate more intelligently with the profession, both as individuals in matters of personal hygiene and as a people in accomplishing sanitary reforms.

